# Excess Length of Acute Inpatient Stay Attributable to Acquisition of Hospital-Onset Gram-Negative Bloodstream Infection with and without Antibiotic Resistance: A Multistate Model Analysis

**DOI:** 10.3390/antibiotics9020096

**Published:** 2020-02-23

**Authors:** Hiroyuki Suzuki, Eli N Perencevich, Rajeshwari Nair, Daniel J Livorsi, Michihiko Goto

**Affiliations:** 1 Center for Access & Delivery Research & Evaluation (CADRE), Iowa City Veterans Affairs Health Care System, Iowa City, IA 52246, USA; hiroyuki-suzuki@uiowa.edu (H.S.); eli-perencevich@uiowa.edu (E.N.P.); rajeshwari-nair@uiowa.edu (R.N.); daniel-livorsi@uiowa.edu (D.J.L.); 2 Department of Internal Medicine, University of Iowa Carver College of Medicine, Iowa City, IA 52242, USA

**Keywords:** multistate model, length of stay, hospital-onset bacteremia, healthcare-associated infection, antimicrobial resistance

## Abstract

Excess length of stay (LOS) is an important outcome when assessing the burden of nosocomial infection, but it can be subject to survival bias. We aimed to estimate the change in LOS attributable to hospital-onset (HO) *Escherichia coli*/*Klebsiella* spp. bacteremia using multistate models to circumvent survival bias. We analyzed a cohort of all patients with HO *E.* *coli*/*Klebsiella* spp. bacteremia and matched uninfected control patients within the U.S. Veterans Health Administration System in 2003–2013. A multistate model was used to estimate the change in LOS as an effect of the intermediate state (HO-bacteremia). We stratified analyses by susceptibilities to fluoroquinolones (fluoroquinolone susceptible (FQ-S)/fluoroquinolone resistant (FQ-R)) and extended-spectrum cephalosporins (ESC susceptible (ESC-S)/ESC resistant (ESC-R)). Among the 5964 patients with HO bacteremia analyzed, 957 (16.9%) and 1638 (28.9%) patients had organisms resistant to FQ and ESC, respectively. Any HO *E.coli*/*Klebsiella* bacteremia was associated with excess LOS, and both FQ-R and ESC-R were associated with a longer LOS than susceptible strains, but the additional burdens attributable to resistance were small compared to HO bacteremia itself (FQ-S: 12.13 days vs. FQ-R: 12.94 days, difference: 0.81 days (95% CI: 0.56–1.05), *p* < 0.001 and ESC-S: 11.57 days vs. ESC-R: 16.56 days, difference: 4.99 days (95% CI: 4.75–5.24), *p* < 0.001). Accurate measurements of excess attributable LOS associated with resistance can help support the business case for infection control interventions.

## 1. Introduction

Healthcare-associated infection (HAI) is an important cause of increased mortality and morbidity in the United States (U.S.) with an estimated annual incidence of 1.7 million cases, with 99,000 deaths and an economic impact of approximately 6.5 billion U.S. dollars [1]. Various strategies to decrease HAIs, including nationwide HAI tracking and bundled infection prevention approaches, have helped reduce the incidence of HAIs to some extent, although it remains a substantial problem in the United States [2].

The recent rise of antimicrobial resistance (AMR) is adding to the health and economic burden of HAIs. In 2019, the Centers for Disease Control and Prevention (CDC) estimated that infections with AMR affect at least 2.8 million people annually and cause at least 35,900 excessive deaths per year in the United States [3]. HAIs with AMR are generally more difficult to treat and associated with increased mortality and morbidity. Studies assessing the mortality and non-mortality burden of AMR are needed because they will help us determine the most cost-effective target in infection control and antimicrobial stewardship, two major strategies to combat HAI and AMR.

Studies have revealed that the mortality from Gram-negative rod (GNR) infections caused by extended-spectrum beta-lactamases (ESBLs) and fluoroquinolone (FQ)-resistant organisms was higher than the mortality from GNR infections caused by susceptible organisms [4,5,6]. In contrast, studies assessing the impact of these resistant organisms on outcomes other than mortality, such as length of stay (LOS), are relatively sparse. Previous observational studies reported that third-generation cephalosporin resistance and fluoroquinolone resistance (FQ-R) in GNR bacteremia were associated with additional LOS when compared to susceptible strains [7,8]. While these studies provided insights into the non-mortality burden of bacteremia attributable to drug resistance, the estimates must be interpreted with caution as the measurement of excess LOS can be subject to survival bias due to competing risks (death or discharge as competing events) and time-dependent bias due to variable timings of disease onset [9,10]. These problems are particularly important when considering the burden of conditions with high mortality, such as bacteremia.

Multistate models have been increasingly used to describe attributable LOS due to drug-resistant organisms in several European studies to account for competing outcomes and time-dependent biases [11,12,13]. These studies found that either ESBL-positive or third-generation cephalosporin resistance were associated with 1.6–9.4 days excess LOS compared with ESBL-negative or third-generation cephalosporin-susceptible strains. One retrospective study by Naylor et al. also found that ciprofloxacin resistance was not associated with excess LOS [13]. However, it was unclear whether these results from Europe were generalizable to the United States, where the healthcare delivery system is substantially different.

The Veterans Health Administration (VHA), the largest integrated health care system in the United States, has aggregated microbiology results nationally. We previously conducted a matched cohort study of hospital-onset (HO) *Escherichia coli* and *Klebsiella* spp. bacteremia to assess the attributable mortality due to FQ-R and extended-spectrum cephalosporin resistance (ESC-R) [4]. In this study, we aimed to quantify the attributable LOS due to HO *E.coli*/*Klebsiella* spp. bacteremia and additional attributable LOS due to FQ-R and ESC-R, using multistate models without considering death as a censoring event.

## 2. Results

During the study period, 5964 patients had HO bloodstream infection (BSI) due to *E. coli* (2663/44.7%) or *Klebsiella* spp. (3301/55.3%). There were 15,213 uninfected control patients matched to the cases and included for analyses. Table 1 describes the characteristics of the study cohort. The characteristics of cohorts with each resistance pattern and their matched uninfected controls are shown in Supplementary Tables S1 and S2. Among case patients, 957 patients (16.9%) and 1638 patients (28.9%) had organisms resistant to FQ and extended-spectrum cephalosporin (ESC), respectively.

Crude total LOS had right-skewed distributions in both groups of case patients and uninfected control patients (case patients: median 23.0 days and mean 62.0 days; control patients: median: 16.0 days and mean 26.3 days; Table 2). Inpatient mortality was substantially higher in the case patient group compared to the uninfected control group (29.1% vs. 9.7%; *p* < 0.0001). The difference in means between the two groups was substantially larger than the difference in medians because of the small number of patients with very prolonged LOS. When stratified by resistance profiles of case patients, both FQ-R and ESC-R were associated with a longer LOS compared to susceptible strains.

After accounting for survival and time-dependent biases, the multistate model estimated the excessive LOS attributable to HO *E. coli*/*Klebsiella* spp. bacteremia as 12.05 days (95% CI: 6.97–17.5) (Table 2). In stratified analyses by isolate susceptibilities to FQ ( FQ-S and FQ-R) and ESC (ESC-S and ESC-R), AMR was associated with a larger change in LOS for both FQ (FQ-S: 12.13 days (95% CI: 6.25–17.88 days) vs. FQ-R: 12.94 days (95% CI: 2.35–24.31 days)) and ESC (ESC-S: 11.57 days (95% CI: 6.25–17.42 days) vs. ESC-R: 16.56 days (95% CI: 3.63–30.38 days)) (Table 2). The difference between attributable LOS per case due to FQ-R was 0.81 days (95% CI: 0.56–1.05 days, *p* < 0.0001) and that due to ESC-R was 4.99 days (95% CI: 4.75–5.24 days, *p* < 0.0001) (Table 3).

Using the multistate model, we calculated the burden of extra LOS due to HO *E. coli*/*Klebsiella* spp. bacteremia during the study period as 14.60 days per 10,000 patient-days (95% CI: 8.44–21.13 days/10,000 patient-days). In contrast, extra LOS due to FQ-R and ESC-R were calculated as 0.17 days per 10,000 patient-days (95% CI: 0.12–0.23 days/10,000 patient-days) and 0.97 days per 10,000 patient-days (95% CI: 0.94–1.00 days/10,000 patient-days), respectively (Table 3).

## 3. Discussion

In this VHA-based retrospective cohort study using a multistate model, we estimated the attributable extra LOS associated with incident HO *E. coli*/*Klebsiella* spp. bacteremia as approximately 12 days. When stratified by isolate susceptibilities to FQ and ESC, attributable LOS associated with ESC-R compared to ESC-S was about five days, and FQ-R was associated with less than one day of attributable LOS. Extra LOS due to all HO *E. coli*/*Klebsiella* spp. bacteremia was 14.6 days per 10,000 days, but the presence of FQ-R and ESC-R was only less than one day extra LOS per 10,000 patient-days. These findings suggest that horizontal infection control measures towards the prevention of HO GNR bacteremia, such as bundles to prevent catheter-associated bloodstream infection or ventilator-associated pneumonia, would be the most effective strategy to decrease the burden from HAIs and AMR because they prevent HAIs irrespective of the resistance pattern.

This is the first United-States-based study to estimate excess LOS of HO bacteremia due to Enterobacteriaceae using a multistate model. Additionally, the use of patient-level matched uninfected controls in our study enabled us to address the true attributable LOS due to antimicrobial resistance without comparing directly between different resistance patterns, which would have different sample characteristics. Our study only included patients with HO bacteremia, thus our results about excess attributable LOS can be interpreted as a reasonable estimate of the non-mortality burden of HAIs.

Our results were comparable with previous studies from Europe, which addressed the burden of antimicrobial resistance and suggest similar impacts of HAI and AMR despite differences in healthcare systems between the United States and European nations. The recent study conducted in the United Kingdom demonstrated that the excess length of stay in patients with *E. coli* bacteremia was 3.87 days (95% CI: 3.69–4.04 days) compared to non-infected control patients, and there were 1.58 days (95% CI: 0.84–2.31 days) of additional excess LOS caused by third-generation cephalosporin resistance [13]. In contrast to third-generation cephalosporin resistance, ciprofloxacin resistance did not significantly prolong LOS (0.46 days, 95% CI: -0.11–1.03 days). The trends in the degree of LOS attributable to the presence of bacteremia and addition from the presence of ESC-R/FQ-R were similar to those of our study, although the absolute excess LOS was much longer in our study. The possible explanation for this absolute difference in LOS is that the previous study included both community-onset and HO infections, and HO infections accounted for only 20% of all cases, while our study only included HO infections. HO sepsis is known to be associated with higher mortalities and a longer LOS compared to community-onset sepsis [14].

As with previous studies addressing the burden of bloodstream infections caused by ESBL-producing or ESC-R Enterobacteriaceae [11,12,13], ESC-R was associated with about five days excess LOS compared with ESC-susceptible strains in our study. There are several possible explanations for the increased LOS caused by ESC-R Enterobacteriaceae. First, patients with bloodstream infections caused by ESBL-producing Enterobacteriaceae are more likely to receive inappropriate empiric antibiotics compared with patients with infections caused by susceptible strains. Inappropriate empiric treatment for bloodstream infections caused by ESBL-producing Enterobacteriaceae is an independent risk factor for mortality [15], and we could assume that patients who survived would have more organ dysfunction, which could prolong LOS. Second, patients with bloodstream infections caused by ESBL-producing Enterobacteriaceae have more serious illnesses and/or comorbidities, which would lead to higher mortality, and when patients survive the episode, to a longer LOS compared with patients with susceptible bacteremia. It is also possible that patients who survived the serious infection may need more healthcare support, such as admission to nursing homes or rehabilitation hospitals, hemodialysis, and wound care. Patients who need such care would require more intensive care coordination before discharge, which could potentially be associated with excess LOS.

Interestingly, FQ-R was only associated with less than one day of excess LOS in our study. In the United States, the majority of hospitals can offer outpatient parenteral antimicrobial therapy (OPAT) when there is no oral antibiotic to switch upon hospital discharge, so the patient does not necessarily have to stay until the completion of therapy. Additionally, it is not common to choose FQ as empiric therapy in hospitalized settings unless a patient has severe beta-lactam allergy. The appropriateness of empiric therapy would not be affected much by the presence of FQ-R unless there is an additional resistance to other classes of antibiotics.

As stated above, our results also highlight the importance of infection prevention as a measure to decrease the burden of HO *E. coli*/*Klebsiella* spp. bacteremia, by demonstrating substantial extra LOS even without antimicrobial resistance, and the presence or absence of resistance to FQ or ESC had relatively small impacts. Infection control measures are generally classified as vertical approaches and horizontal approaches [16]. While vertical approaches utilize activities that are directed at a single type of pathogen or resistance pattern, horizontal approaches, such as hand hygiene or prevention bundles for catheter-related bloodstream infection, aim to reduce the risk of infections due to a broad array of pathogens irrespective of resistance pattern [17]. Our results call for more attention towards horizontal infection control measures rather than vertical measures, which typically target only specific pathogens or resistant organisms. Antimicrobial stewardship, especially focusing on minimizing unnecessary ESC use, can have an additional benefit by preventing additional burden due to antimicrobial resistance.

Our study has several limitations. First, we could not obtain detailed patient-level clinical information such as the source of bacteremia, time to receipt of appropriate antimicrobial therapy, presence/absence of surgery, infectious diseases consultation, intensive care unit admission, consequences of bacteremia, or patient disposition upon hospital discharge. Second, antimicrobial resistance was only assessed by phenotypic reports from clinical microbiology labs, as we did not have detailed microbiologic information such as strain types or mechanisms of antimicrobial resistance. Third, we could not subcategorize our cohort by species or combinations of antimicrobial resistance patterns [4], because we did not have large enough sample sizes. As the multistate model simultaneously estimates five hazards with time-dependent covariate (HO bacteremia), it requires large sample sizes to achieve enough statistical power to detect significant differences. Fourth, while we accounted for the important basic patient demographics (age, gender, and season), the admission facility, and the most important sources of biases (survival bias and time-dependent bias), it was not technically feasible to account for comorbidities or potential nonproportional hazards because of the lack of an established statistical methodology. Fifth, this study only included two species of Enterobacteriaceae, and further study is needed to estimate the total burden of HO bacteremia as a whole. Finally, it is unclear how this result may apply beyond the studied VHA population.

## 4. Materials and Methods

### 4.1. Ethics

The institutional review board (IRB) of the University of Iowa and Research & Development Committee of the Iowa *City* Veterans Affairs Health Care System approved this study (the ethical approval number IRB is #201706750). The IRB granted a waiver for informed consent for this retrospective cohort.

### 4.2. Study Population and Study Design

We used a patient-level matched cohort design, using uninfected patients as the control group [4,18]. Case patients were all veterans admitted to acute-care units at VHA hospitals in 48 continental states and the District of Columbia in the United States between January 2003 and December 2013 and who had first positive blood cultures for *E. coli* or *Klebsiella* spp. at least 48 hours after admission. If a patient had multiple positive blood cultures for the same species during the same hospital admission, we included only the first isolate in the analysis. We selected uninfected controls who were matched at the patient-level based on sex, the month of admission, and the facility. Also, the length of stay of uninfected controls was matched to the length of stay before the onset of bacteremia of case-patients [19]. For each case, up to three uninfected controls were selected without replacement.

### 4.3. Data Source

We obtained data from the Corporate Data Warehouse (CDW) through the Veterans Affairs Informatics and Computing Infrastructure (VINCI), which includes data extracted from VHA’s integrated electronic medical record system [20]. Susceptibility results in microbiology reports were recorded in a standardized manner, and isolates were classified as not susceptible if they were reported as intermediate resistance or resistant. Minimal inhibitory concentrations (MICs) or sizes of inhibition zones were not typically available.

### 4.4. Definition of Variables

Comorbidities were assessed using diagnosis codes using the Charlson method based on administrative data [21]. HO bacteremia was defined as the first positive blood culture taken after the patient had been in the hospital for 48 hours or longer. FQ-R was defined as a non-susceptible result to at least one of the FQs: ciprofloxacin, levofloxacin, or moxifloxacin. ESC-R was defined as a non-susceptible result to at least one the following: ceftriaxone, ceftazidime, or cefepime.

### 4.5. Outcome

Our primary outcome measure was LOS after the first positive blood culture in case (bacteremic) patients. For uninfected control patients, we subtracted the LOS before bacteremia onset of the matched case patients from the total LOS, to allow us to estimate the difference of the expected subsequent stay given the presence or absence of infection. We also measured the vital status at hospital discharge (death vs. discharge alive) as the terminal states of the multistate model (see Statistical Analysis section).

### 4.6. Statistical Analysis

Patient characteristics were compared between cases and controls using Fisher’s exact test for categorical variables and the Mann–Whitney U test for continuous variables.

**Figure 1:**
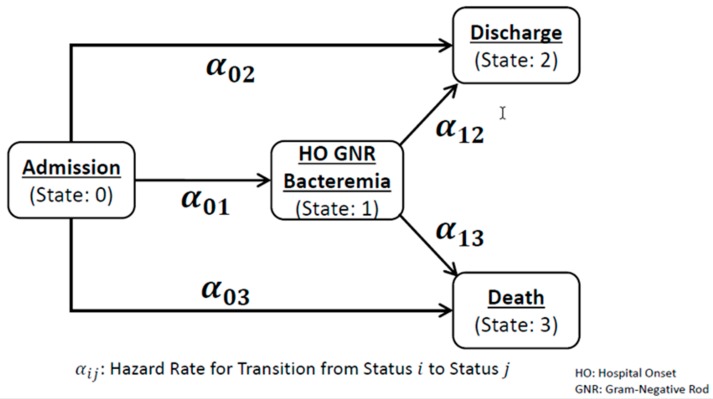
Diagram of the Multistate Model to Estimate Extra Length of Stay.

We used a multistate model to estimate the change in LOS as an effect of the intermediate state (HO *E. coli*/*Klebsiella* spp. bacteremia; Figure 1) [22,23]. In brief, the multistate model accounts for the time-dependent exposure (occurrence of HO *E. coli*/*Klebsiella* spp. bacteremia) and competing endpoints (hospital discharge or death), as illustrated in Figure 1. In this study, we designed four states (state 0—admission without bacteremia; state 1—acquisition of bacteremia; state 2—discharged alive from acute care hospital; state 3—death at the discharge) with five one-directional hazards ($\alpha_{01}$—hazard rate of acquiring bacteremia; $\alpha_{02}$—hazard rate of discharge alive without acquiring bacteremia;$\;\alpha_{03}$—hazard rate of death without acquiring bacteremia;$\;\alpha_{12}$—hazard rate of discharge alive after acquiring bacteremia;$\;\alpha_{13}$—hazard rate of death after acquiring bacteremia), where $\alpha_{12}$ and $\alpha_{13}$ are conditioned to the acquisition of HO bacteremia due to *E. coli* or *Klebsiella* spp. We assumed constant hazards during the inpatient stay and estimated the matrix of hazards simultaneously by a time-inhomogeneous model using the Aalen–Johansen estimator while accounting for HO bacteremia as a time-dependent covariate [24,25,26,27]. This matrix of hazards (transition probabilities from one state to another) allowed us to perform nonparametric estimation of extra LOS by methods proposed by Schulgen and Schumacher [25]. We first analyzed HO bacteremia due to *E. coli* or *Klebsiella* spp. without considering AMR, then performed stratified analyses by resistance profiles to (FQ susceptible (FQ-S) vs. FQ-R and extended-spectrum cephalosporin susceptible (ESC-S) vs. ESC-R). We estimated 95% confidence intervals (CIs) of extra LOS by bootstrapping.

We estimated the excess LOS per case, as well as per 10,000 patient-days by multiplying the LOS per case times the cumulative incidence (total excessive LOS within the VHA system), divided by total patient-days of acute care units within the VHA system. Finally, a comparison of excess LOS between FQ-S/FQ-R and ESC-S/ESC-R was performed using the Mann–Whitney U test. All statistical analyses were performed on R version 3.6.1 (R Foundation for Statistical Computing, Vienna, Austria) with *etm* package version 1.0.5 [27].

## 5. Conclusions

This VHA-based retrospective cohort study using a multistate model estimated that the attributable LOS of patients associated with HO *E. coli*/*Klebsiella* spp. bacteremia was about 12 days. Additional excess LOS was associated with resistance compared to susceptible strains, but the impact was not as large as that of HO bacteremia itself. These findings could inform future business-case analyses in support of infection control or antimicrobial stewardship interventions.

## Figures and Tables

**Table 1:** Characteristics of cohort patients and uninfected control patients (2003–2013).

Variables	HO Bacteremia Case Patients (*n* = 5964)	Uninfected Control Patients (*n* = 15,213)	*p*-Value
Age (mean (SD))	68.1 (12.1)	68.2 (12.0)	0.76
Male Gender (%)	5834 (97.8)	14,902 (98.0)	0.57
BMI (mean (SD))	26.1 (7.2)	26.4 (7.2)	0.003
GNR Species			
*Escherichia coli*	2663 (44.7)	N/A	N/A
*Klebsiella* spp.	3301 (55.3)	N/A	N/A
Charlson Comorbidities			
Myocardial Infarction (%)	828 (13.9)	2479 (16.3)	<0.001
Congestive Heart Failure (%)	1430 (24.0)	4656 (30.6)	<0.001
Peripheral Vascular Disease (%)	905 (15.2)	3784 (24.9)	<0.001
Cerebrovascular Disease (%)	1031 (17.3)	3513 (23.1)	<0.001
Dementia (%)	307 (5.1)	920 (6.0)	0.01
Chronic Pulmonary Disease (%)	1596 (26.8)	5948 (39.1)	<0.001
Rheumatic Disease (%)	144 (2.4)	430 (2.8)	0.11
Peptic Ulcer (%)	355 (6.0)	917 (6.0)	0.86
Mild Liver Disease (%)	995 (16.7)	2055 (13.5)	<0.001
Moderate/Severe Liver Disease (%)	351 (5.9)	530 (3.5)	<0.001
Diabetes with No Chronic Complication (%)	2210 (37.1)	6443 (42.4)	<0.001
Diabetes with Chronic Complication (%)	681 (11.4)	2838 (18.7)	<0.001
Hemiplegia/Paraplegia (%)	467 (7.8)	827 (5.4)	<0.001
Renal Disease (%)	1447 (24.3)	3845 (25.3)	0.13
Malignancy (%)	2128 (35.7)	4809 (31.6)	<0.001
Metastatic Tumor (%)	733 (12.3)	1448 (9.5)	<0.001
HIV/AIDS (%)	83 (1.4)	255 (1.7)	0.15
Outcomes			
Inpatient Mortality	1737 (29.1)	1480 (9.7)	<0.001
30-day Mortality	1623 (27.2)	2023 (13.3)	<0.001
90-day Mortality	2351 (39.4)	3513 (23.1)	<0.001

HO—Hospital-onset, SD—Standard deviation, BMI—Body mass index, GNR—Gram-negative rod, HIV—Human immunodeficiency virus, AIDS—Acquired immunodeficiency syndrome.

**Table 2:** Excess length of stay caused by hospital-onset *Escherichia coli*/*Klebsiella* spp. bacteremia, stratified by the presence/absence of resistance patterns.

	*Case Patients*		*Uninfected Controls*			
Resistance Patterns	Median/Mean LOS (Days (IQR))	Inpatient Mortality	Median/Mean LOS (Days (IQR))	Inpatient Mortality	Difference of Median/Mean of LOS (Days)	Extra LOS per Case from Multistate Model (Days (95% CI))
HO *E. coli*/*Klebsiella* spp. Bacteremia (Overall)	23.0/62.0 (12.0–46.0)	29.1%	16.0/26.3 (8.0–31.0)	9.7%	7.0/35.7	12.05 (6.97–17.5)
Stratified Analysis by Fluoroquinolone Resistance Profile						
FQ-S	21.0/56.3 (12.0–42.0)	25.3%	14.0/23.6 (8.0–28.0)	8.9%	7.0/32.7	12.13 (6.25–17.88)
FQ-R	30.0/77.2 (16.0–59.0)	39.1%	21.0/33.5 (10.0–39.0)	12.0%	9.0/43.7	12.94 (2.35–24.31)
Stratified Analysis by Extended-Spectrum Cephalosporin Resistance Profile						
ESC-S	22.0/58.0 (12.0–43.0)	26.9%	15.0/24.2 (8.0–28.0)	9.0%	7.0/33.8	11.57 (6.25–17.42)
ESC-R	33.0/83.1 (18.0–64.0)	44.7%	24.0/37.2 (12.0–45.0)	13.6%	9.0/45.9	16.56 (3.63–30.38)

LOS—Length of stay, IQR—Interquartile range, CI—Confidence interval, HO—Hospital-onset, FQ-S—Fluoroquinolone susceptible, FQ-R—Fluoroquinolone resistant, ESC-S—Extended-spectrum cephalosporin susceptible, ESC-R—Extended-spectrum cephalosporin resistant.

**Table 3:** Burden of extra length of stay due to hospital-onset *Escherichia coli*/*Klebsiella* spp. bacteremia and fluoroquinolone/extended-spectrum cephalosporin resistance, per 10,000 patient-days within the Veterans Health Administration (VHA) System (2003–2013).

Resistance patterns	Total Burden of Extra LOS Due to HO *E. coli/Klebsiella* spp. Bacteremia (Days (95% CI))	Extra LOS Due to HO *E. coli/Klebsiella* spp. Bacteremia per Patient-Days (Days/10,000 Patient-Days (95% CI))	Additional Extra LOS Due to Resistance (Days (95% CI))	Total Burden of Extra LOS Due to Resistance among HO *E. coli/Klebsiella* spp. bacteremia (Days (95% CI))	Extra LOS Due to Resistance among HO *E. coli/Klebsiella* spp. Bacteremia per Patient-Days (Days/10,000 Patient-Days (95% CI))
FQ-R	71890.1 (41,588.9–104,098.8)	14.60 (8.44–21.13)	0.81 (0.57–1.05)	855.8 (598.6–1113.2)	0.17 (0.12–0.23)
ESC-R			4.99 (4.85–5.13)	4776.4 (4640.9–4905.6)	0.97 (0.94–1.00)

LOS—Length of stay, CI—Confidence interval, HO—Hospital-onset, FQ-R—Fluoroquinolone resistant, ESC-R—Extended-spectrum cephalosporin resistant.

## Supplementary Materials

The following are available online at https://www.mdpi.com/2079-6382/9/2/96/s1, Table S1: Characteristics of Included Patients Stratified by Susceptibility to Fluoroquinolone, Table S2: Characteristics of Included Patients Stratified by Susceptibility to Extended-Spectrum Cephalosporin.
