# Supplementary material for: Excess Length of Acute Inpatient Stay Attributable to Acquisition of Hospital-Onset Gram-Negative Bloodstream Infection with and without Antibiotic Resistance: A Multistate Model Analysis
Source: Antibiotics (Basel). 2020 Feb 23;9(2):96. doi: 10.3390/antibiotics9020096 (PMC7168210; doi:10.3390/antibiotics9020096)
Supplement: Supplementary file 1 [file antibiotics-09-00096-s001.pdf]

Supplementary Table 1. Characteristics of Included Patients Stratified by Susceptibility to Fluoroquinolone

	**Fluoroquinolone Susceptible**			**Fluoroquinolone Resistant**		
	Case Patients with HO bacteremia (n=4,326)	Uninfected Control Patients (n=11,042)	p-value	Case Patients with HO bacteremia (n=1,638)	Uninfected Control Patients (n=4,171)	p-value
**Age (mean (SD))**	67.8 (12.0)	68.0 (11.9)	0.43	69.0 (12.5)	68.8 (12.0)	0.5
**Male Gender (%)**	4,223 (97.6)	10,797 (97.8)	0.59	1,611 (98.4)	4,105 (98.4)	0.95
**BMI (mean (SD))**	26.0 (7.2)	26.6 (7.2)	<0.001	26.3 (7.1)	26.0 (7.2)	0.17
**GNR Species**						
***Escherichia coli* (%)**	1,702 (39.3)	N/A	N/A	961 (58.7)	N/A	N/A
***Klebsiella* spp.**	2,624 (60.7)	N/A	N/A	677 (41.3)	N/A	N/A
**Resistant to ES Cephalosporin (%)**	199 (4.6)	N/A	N/A	758 (46.3)	N/A	N/A
**Charlson Comorbidities**						
**Myocardial Infarction (%)**	558 (12.9)	1,767 (16.0)	<0.001	270 (16.5)	712 (17.1)	0.62
**Congestive Heart Failure (%)**	952 (22.0)	3,349 (30.3)	<0.001	478 (29.2)	1,307 (31.3)	0.12
**Peripheral Vascular Disease (%)**	628 (14.5)	2,737 (24.8)	<0.001	277 (16.9)	1,047 (25.1)	<0.001
**Cerebrovascular Disease (%)**	693 (16.0)	2,528 (22.9)	<0.001	338 (20.6)	985 (23.6)	0.02
**Dementia (%)**	183 (4.2)	627 (5.7)	<0.001	124 (7.6)	293 (7.0)	0.50
**Chronic Pulmonary Disease (%)**	1,117 (25.8)	4,396 (39.8)	<0.001	479 (29.2)	1,552 (37.2)	<0.001
**Rheumatic Disease (%)**	96 (2.2)	321 (2.9)	0.02	48 (2.9)	109 (2.6)	0.56
**Peptic Ulcer (%)**	259 (6.0)	663 (6.0)	1.00	96 (5.9)	254 (6.1)	0.79
**Mild Liver Disease (%)**	699 (16.2)	1,477 (13.4)	<0.001	296 (18.1)	578 (13.9)	<0.001
**Moderate to Severe Liver Disease (%)**	246 (5.7)	399 (3.6)	<0.001	105 (6.4)	131 (3.1)	<0.001
**Diabetes with No Chronic Complication (%)**	1,518 (35.1)	4,623 (41.9)	<0.001	692 (42.2)	1820 (43.6)	0.35
**Diabetes with Chronic Complication (%)**	442 (10.2)	2,014 (18.2)	<0.001	239 (14.6)	824 (19.8)	<0.001
**Hemiplegia/Paraplegia (%)**	245 (5.7)	596 (5.4)	0.54	222 (13.6)	231 (5.5)	<0.001
**Renal Disease (%)**	943 (21.8)	2,761 (25.0)	<0.001	504 (30.8)	1,084 (26.0)	<0.001
**Malignancy (%)**	1,597 (36.9)	3,412 (30.9)	<0.001	531 (32.4)	1,397 (33.5)	0.45
**Metastatic Tumor (%)**	579 (13.4)	1,032 (9.3)	<0.001	154 (9.4)	416 (10.0)	0.54
**HIV/AIDS (%)**	52 (1.2)	167 (1.5)	0.17	31 (1.9)	88 (2.1)	0.67
**Outcomes**						
**Inpatient Mortality (%)**	1,096 (25.3)	978 (8.9)	<0.001	641 (39.1)	502 (12.0)	<0.001
**30-day Mortality (%)**	1,060 (24.5)	1,401 (12.7)	<0.001	563 (34.4)	622 (14.9)	<0.001
**90-day Mortality (%)**	1,556 (36.0)	2,422 (21.9)	<0.001	795 (48.5)	1091 (26.2)	<0.001

HO: Hospital-onset, BMI: Body mass index, GNR: Gram-negative rod, HIV: Human immunodeficiency virus, AIDS: Acquired immunodeficiency syndrome

Supplementary Table 2. Characteristics of Included Patients Stratified by Susceptibility to Extended-Spectrum Cephalosporin

	ES Cephalosporin Susceptible			ES Cephalosporin Resistant		
	Case Patients with HO bacteremia (n=5,007)	Uninfected Control Patients (n=12,764)	p-value	Case Patients with HO bacteremia (n=957)	Uninfected Control Patients (n=2,449)	p-value
**Age (mean (SD))**	67.9 (12.0)	68.0 (11.9)	0.97	69.1 (12.6)	69.4 (12.0)	0.52
**Male Gender (%)**	4,894 (97.7)	12,497 (97.9)	0.53	940 (98.2)	2,405 (98.2)	1.00
**BMI (mean (SD))**	25.9 (7.2)	26.6 (7.2)	<0.001	26.7 (7.3)	25.6 (7.0)	<0.001
**GNR Species**						
***Escherichia coli* (%)**	2,410 (48.1)	N/A	N/A	253 (26.4)	N/A	N/A
***Klebsiella* spp.**	2,597 (51.9)	N/A	N/A	704 (53.6)	N/A	N/A
**Resistant to Fluoroquinolone (%)**	880 (17.6)	N/A	N/A	758 (79.2)	N/A	N/A
**Charlson Comorbidities**						
**Myocardial Infarction (%)**	652 (13.0)	2,030 (15.9)	<0.001	176 (18.4)	449 (18.3)	1.00
**Congestive Heart Failure (%)**	1,133 (22.6)	3,889 (30.5)	<0.001	297 (31.0)	767 (31.3)	0.91
**Peripheral Vascular Disease (%)**	723 (14.4)	3,124 (24.5)	<0.001	182 (19.0)	660 (26.9)	<0.001
**Cerebrovascular Disease (%)**	821 (16.4)	2,880 (22.6)	<0.001	210 (21.9)	633 (25.8)	0.02
**Dementia (%)**	232 (4.6)	694 (5.4)	0.03	75 (7.8)	226 (9.2)	0.22
**Chronic Pulmonary Disease (%)**	1,321 (26.4)	5,080 (39.8)	<0.001	275 (28.7)	868 (35.4)	<0.001
**Rheumatic Disease (%)**	122 (2.4)	363 (2.8)	0.15	22 (2.3)	67 (2.7)	0.55
**Peptic Ulcer (%)**	291 (5.8)	764 (6.0)	0.69	64 (6.7)	153 (6.2)	0.69
**Mild Liver Disease (%)**	835 (16.7)	1,701 (13.3)	<0.001	160 (16.7)	354 (14.5)	0.11
**Moderate to Severe Liver Disease (%)**	293 (5.9)	443 (3.5)	<0.001	58 (6.1)	87 (3.6)	0.002
**Diabetes with No Chronic Complication (%)**	1,814 (36.2)	5,344 (41.9)	<0.001	396 (41.4)	1,099 (44.9)	0.07
**Diabetes with Chronic Complication (%)**	527 (10.5)	2,348 (18.4)	<0.001	154 (16.1)	490 (20.0)	0.01
**Hemiplegia/Paraplegia (%)**	315 (6.3)	691 (5.4)	0.03	152 (15.9)	136 (5.6)	<0.001
**Renal Disease (%)**	1,144 (22.8)	3,186 (25.0)	0.003	303 (31.7)	659 (26.9)	0.01
**Malignancy (%)**	1,864 (37.2)	3,941 (30.9)	<0.001	264 (27.6)	868 (35.4)	<0.001
**Metastatic Tumor (%)**	655 (13.1)	1,189 (9.3)	<0.001	78 (8.2)	259 (10.6)	0.04
**HIV/AIDS (%)**	66 (1.3)	193 (1.5)	0.37	17 (1.8)	62 (2.5)	0.23
**Outcomes**						
**Inpatient Mortality (%)**	1,309 (26.1)	1,148 (9.0)	<0.001	428 (44.7)	332 (13.6)	<0.001
**30-day Mortality (%)**	1,271 (25.4)	1,642 (12.9)	<0.001	352 (36.8)	381 (15.6)	<0.001
**90-day Mortality (%)**	1,859 (37.1)	2,829 (22.2)	<0.001	492 (51.4)	684 (27.9)	<0.001

HO: Hospital-onset, BMI: Body mass index, GNR: Gram-negative rod, HIV: Human immunodeficiency virus, AIDS: Acquired immunodeficiency syndrome
